# Atherogenic index of plasma is associated with epicardial adipose tissue volume assessed on coronary computed tomography angiography

**DOI:** 10.1038/s41598-022-13479-5

**Published:** 2022-06-10

**Authors:** Jeremy Yuvaraj, Mourushi Isa, Zhu Chung Che, Egynne Lim, Nitesh Nerlekar, Stephen J. Nicholls, Sujith Seneviratne, Andrew Lin, Damini Dey, Dennis T. L. Wong

**Affiliations:** 1 Monash Cardiovascular Research Centre, Victorian Heart Institute, MonashHeart and Monash University, Monash Health, 246 Clayton Road, Clayton, VIC 3168 Australia; 2 School of Clinical Sciences, Monash University, Clayton, VIC Australia; 3 Biomedical Imaging Research Institute, Cedars-Sinai Medical Center, Los Angeles, CA USA

**Keywords:** Dyslipidaemias, Atherosclerosis, Coronary artery disease and stable angina

## Abstract

The atherogenic index of plasma (AIP) is a novel biomarker of atherogenic dyslipidaemia (AD), but its relationship with cardiac adipose tissue depots is unknown. We aimed to assess the association of AD with cardiac adipose tissue parameters on coronary computed tomography angiography (CCTA). We studied 161 patients who underwent CCTA between 2008 and 2011 (age 59.0 ± 14.0 years). AD was defined as triglyceride (TG) > 1.7 mmol/L and HDL < 1.0 mmol/L (n = 34). AIP was defined as the base 10 logarithmic ratio of TG to HDL. Plaque burden was assessed using the CT-Leaman score (CT-LeSc). We studied volume and attenuation of epicardial adipose tissue (EAT-v and EAT-a) and pericoronary adipose tissue (PCAT-v and PCAT-a) on CCTA using semi-automated software. Patients with AD had higher PCAT-v (p = 0.042) and EAT-v (p = 0.041). AIP was associated with EAT-v (p = 0.006), type II diabetes (p = 0.009) and male sex (p < 0.001) and correlated with CT-LeSc (p = 0.040). On multivariable analysis, AIP was associated with EAT-v ≥ 52.3 cm^3^, age, male sex and type II diabetes when corrected for traditional risk factors and plaque burden. AIP is associated with increased EAT volume, but not PCAT-a, after multivariable adjustment. These findings indicate AIP is associated with adverse adipose tissue changes which may increase coronary risk.

## Introduction

Cardiovascular risk is heightened significantly by the presence of metabolic syndrome, a condition featuring a clustering of risk factors including obesity, insulin resistance, hypertension, and dysregulated lipoprotein metabolism. The lattermost condition may be typified by increased triglyceride (TG) and decreased high-density lipoprotein (HDL) cholesterol, the combination of which is termed atherogenic dyslipidaemia (AD) and has been associated with an increased risk of major adverse cardiac events (MACE)^1,2^. The lipid imbalance that characterises this condition has been previously defined as the atherogenic index of plasma (AIP), which describes the logarithmic transformation of the ratio of TG to HDL^3^. This quantitative representation of AD has been associated with coronary artery disease (CAD) burden and event risk^3–6^ even among patients with normal lipid levels in isolation^7^. AIP therefore provides additive value as a quantitative measure of the interaction of TG and HDL rather than simply the numerical values of these parameters, whilst further highlighting the relationship between dyslipidaemia and poorer cardiovascular outcomes.

It is well established that vascular inflammation is intertwined with atherosclerotic risk^8,9^. Vascular inflammation within the coronary arteries effect changes to neighbouring adipocytes detectable on coronary computed tomography angiography (CCTA)^10^, such that adipose tissue enclosed within the pericardial sac, namely epicardial adipose tissue (EAT) and pericoronary adipose tissue (PCAT), are well recognised as independent markers of cardiometabolic risk. Increased EAT is indicative of excess visceral adiposity and metabolic dysfunction^11^ that has been associated with CAD^11–13^. PCAT radiodensity acts as a surrogate measurement of vascular inflammation specifically within the coronary vasculature, and is robustly associated with poor outcomes^10,14,15^. While volumetric increases in EAT may be related to increased TG and decreased HDL^11^, it remains unclear if adverse changes to either adipose tissue depot are associated with dyslipidaemia. Therefore, we aimed to study the relationship between AIP as a quantitative marker of AD severity and changes to the volume and density of cardiac adipose tissue depots.

## Results

### Baseline characteristics

Baseline patient characteristics are summarised in Table 1. A total of 161 patients were studied (age 59.4 ± 14.0 years; 55.3% male). AD was present in 34 patients (21.1%). A higher proportion of AD patients were male (76.5% [n = 26] male vs. 23.5% [n = 8] female, p = 0.005). There were no other significant differences in risk factor prevalence between patients with and without AD (all p > 0.05). For all patients, median TC was 4.5 (IQR 3.9–5.4) mmol/L, TG was 1.2 (IQR 0.9–1.9) mmol/L, HDL-c was 1.0 (IQR 0.9–1.4) mmol/L, and mean LDL-c was 2.83 ± 1.13 mmol/L. Mean AIP for all patients was 0.082 ± 0.319.

**Table 1:** Baseline characteristics.

	AD (n = 34)	No AD (n = 127)	*p* value
AIP, mean ± SD	0.485 ± 0.176	− 0.026 ± 0.255	**< 0.001**
Age, mean ± SD	57.5 ± 12.0	59.9 ± 14.5	0.390
Male, n (%)	26 (76.5)	63 (49.6)	**0.005**
**Cardiovascular risk factors, n (%)**			
Hypertension	22 (64.7)	72 (56.7)	0.400
Smoker	13 (38.2)	51 (40.2)	0.839
Obesity	9 (26.5)	27 (21.3)	0.517
Family history IHD	15 (44.1)	45 (35.4)	0.352
Type II diabetes	9 (26.5)	17 (13.4)	0.066
CAD, n (%)^a^	28 (82.4)	89 (71.2)	0.191
**Plaque burden, n (%)**			0.402
No coronary plaque	6 (17.6)	36 (28.8)	
Low plaque burden	17 (50.0)	57 (45.6)	
High plaque burden	11 (32.4)	32 (25.6)	
CT-LeSc, median (IQR)^a^	3.85 (1.54, 9.12)	3.69 (0.00, 8.52)	0.271
Statin, n (%)^a^	9 (42.9)	24 (33.8)	0.447

Significant values are in bold.

^a^Proportions are of modified totals excluding missing data.

### Atherogenic index of plasma and coronary plaque burden

**Figure 1:**
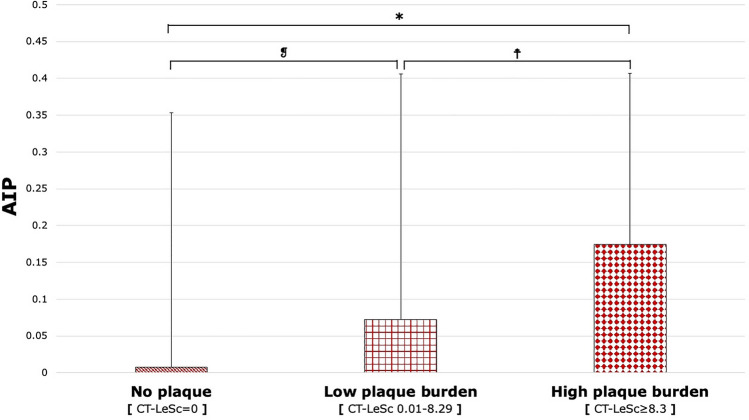
AIP across categories of coronary plaque burden. AIP progressively increased across as plaque burden increased categorically (p = 0.039). AIP was significantly different between no plaque burden (CT-LeSc = 0) and high plaque burden (CT-LeSc ≥ 8.3) subgroups. *p = 0.035; ^❡^p = 0.719; ^**☨**^p = 0.272. *AIP* atherogenic index of plasma, *CT-LeSc* computed tomography-Leaman score.

AIP was numerically, but not significantly, higher in patients with CAD compared to those without CAD (0.113 ± 0.304 vs. 0.008 ± 0.345, p = 0.056; see Supplementary Fig. S1). AIP was weakly correlated with CT-LeSc (r = 0.163, p = 0.040). Furthermore, AIP progressively increased with categories of plaque burden (p = 0.039) and was significantly higher in patients with high plaque burden compared to patients with no plaque (0.175 ± 0.232 vs. 0.008 ± 0.345, p = 0.035; see Fig. 1). Additionally, AIP was numerically, but not significantly, higher in patients with high plaque burden compared to patients with low plaque burden (0.175 ± 0.232 vs. 0.072 ± 0.334, p = 0.272; see Fig. 1).

### Atherogenic index of plasma and cardiometabolic risk

AIP was significantly higher in men (0.158 ± 0.293 vs. − 0.013 ± 0.325, p < 0.001) and patients with type II diabetes (0.231 ± 0.284 vs. 0.053 ± 0.318, p = 0.009); AIP was not associated with any other risk factors (all p > 0.05) and was only numerically higher among patients on statin treatment (0.167 ± 0.294 vs. 0.049 ± 0.313, p = 0.081).

### Cardiac adipose tissue markers and cardiometabolic risk

Mean PCAT-a in the entire cohort was − 67.0 ± 10.1 HU, mean PCAT-v was 1.1 ± 0.5 cm^3^, median EAT-a was -76.7 (IQR − 79.9 to − 73.5) HU and median EAT-v was 52.3 (IQR 37.3–75.5) cm^3^. When assessed against traditional cardiovascular risk factors, PCAT-a was not associated with any risk factors (all p > 0.05). PCAT-v was significantly higher among male subjects (1.2 ± 0.5 cm^3^ vs. 1.0 ± 0.5 cm^3^, p = 0.028). EAT-a was positively associated with BMI (beta = 0.240 [95% CI 0.043–0.437], p = 0.018), and was significantly increased in patients with male sex (− 75.8 [IQR − 79.7 to − 72.7] HU vs. − 77.7 [IQR − 80.2 to − 74.8] HU, p = 0.039), hypertension (− 76.0 [IQR − 78.8 to − 72.9] HU vs. − 78.4 [IQR − 81.7 to − 74.4] HU, p = 0.011), and positive smoking status (− 75.6 [IQR − 79.7 to − 72.4] HU vs. − 77.1 [IQR − 80.0 to − 74.8] HU, p = 0.045). EAT-v was positively associated with age (beta = 0.582 [95% CI 0.247–0.917], p < 0.001) and BMI (beta = 1.948 [95% CI 0.713–3.183], p = 0.002), and was significantly increased in subjects with male sex (63.4 [IQR 40.5–86.5] cm^3^ vs. 48.0 [IQR 34.0–61.9] cm^3^, p = 0.001) and obesity (68.9 [IQR 48.2–90.0] cm^3^ vs. 51.0 [IQR 36.6–68.6] cm^3^, p = 0.007).

### Cardiac adipose tissue markers and atherogenic index of plasma

Adipose tissue changes were first studied in relation to isolated dysregulated lipid parameters. TG correlated with EAT-v (r = 0.170, p = 0.031). HDL-c was inversely correlated with PCAT-v (r = − 0.183, p = 0.035), EAT-a (r = − 0.169, p = 0.032), and EAT-v (r = − 0.297, p < 0.001).

Isolated lipid parameters were also evaluated as binary outcome variables. High TC was not significantly associated with PCAT-a (p = 0.367), PCAT-v (p = 0.394), EAT-a (p = 0.830) or EAT-v (p = 0.663). Likewise, high LDL and low HDL were not significantly associated with PCAT-a, PCAT-v, EAT-a or EAT-v (all p > 0.05). EAT-v was non-significantly higher among patients with high TG (57.0 [40.2–85.4] cm^3^ vs. 51.0 [IQR 35.5–72.1] cm^3^, p = 0.094). High TG was likewise not associated with PCAT-a, PCAT-v or EAT-a (all p > 0.05).

**Figure 2:**
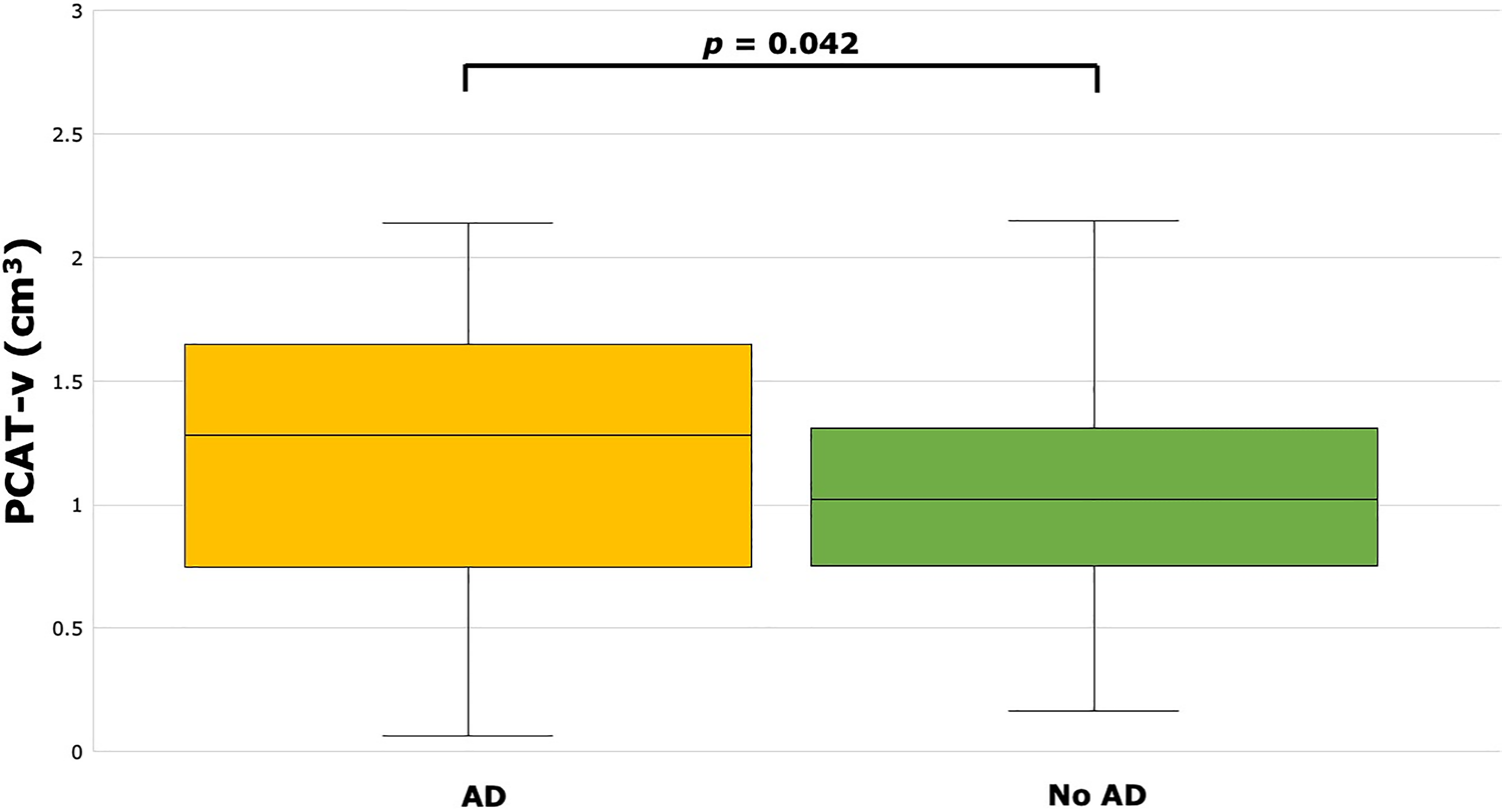
PCAT-v was significantly higher in patients with AD (1.24 ± 0.58 cm^3^) versus patients without AD (1.03 ± 0.45 cm^3^). *PCAT-v* pericoronary adipose tissue volume, *AD* atherogenic dyslipidaemia.

**Figure 3:**
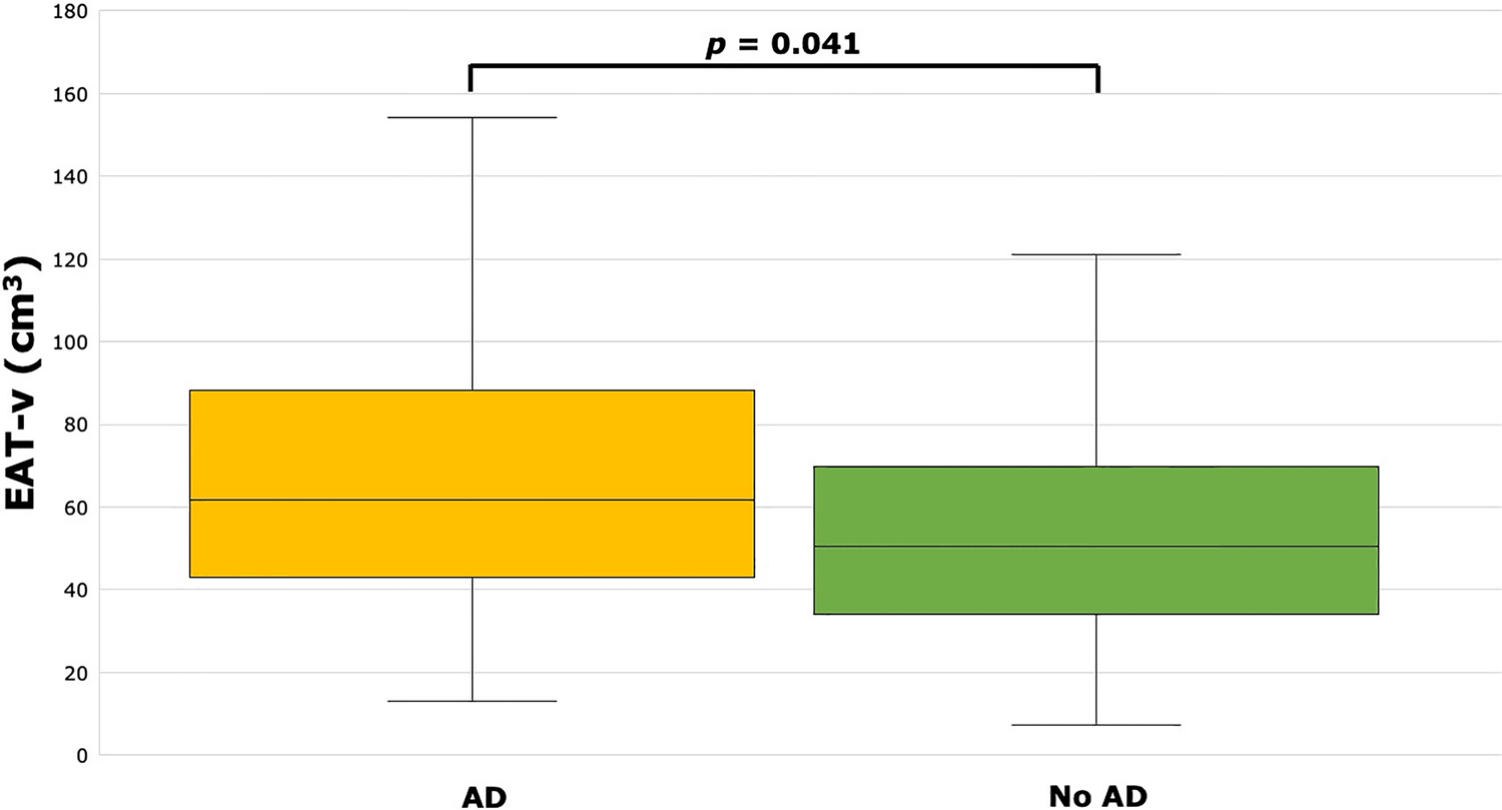
EAT-v was significantly higher in patients with AD (61.7 [IQR 43.0–88.2] cm^3^) versus patients without AD (51.0 [IQR 36.5–70.5] cm^3^). *EAT-v* epicardial adipose tissue volume, *AD* Atherogenic dyslipidaemia.

Patients with both high TG and low HDL (AD) had significantly higher PCAT-v compared to patients with no AD (1.24 ± 0.58 cm^3^ vs. 1.03 ± 0.45 cm^3^, p = 0.042; see Fig. 2). Patients with AD also had significantly higher EAT-v compared to patients with no AD (61.7 [IQR 43.0–88.2] cm^3^ vs. 51.0 [IQR 36.5–70.5] cm^3^, p = 0.041; see Fig. 3). There were no significant differences in EAT-a (− 77.0 [IQR − 79.8 to − 74.1] HU vs. − 76.7 [IQR − 80.0 to − 73.5] HU, p = 0.740) or PCAT-a (− 70.2 ± 10.9 HU vs. − 66.2 ± 9.8 HU, p = 0.068) between patients with and without AD.

**Figure 4:**
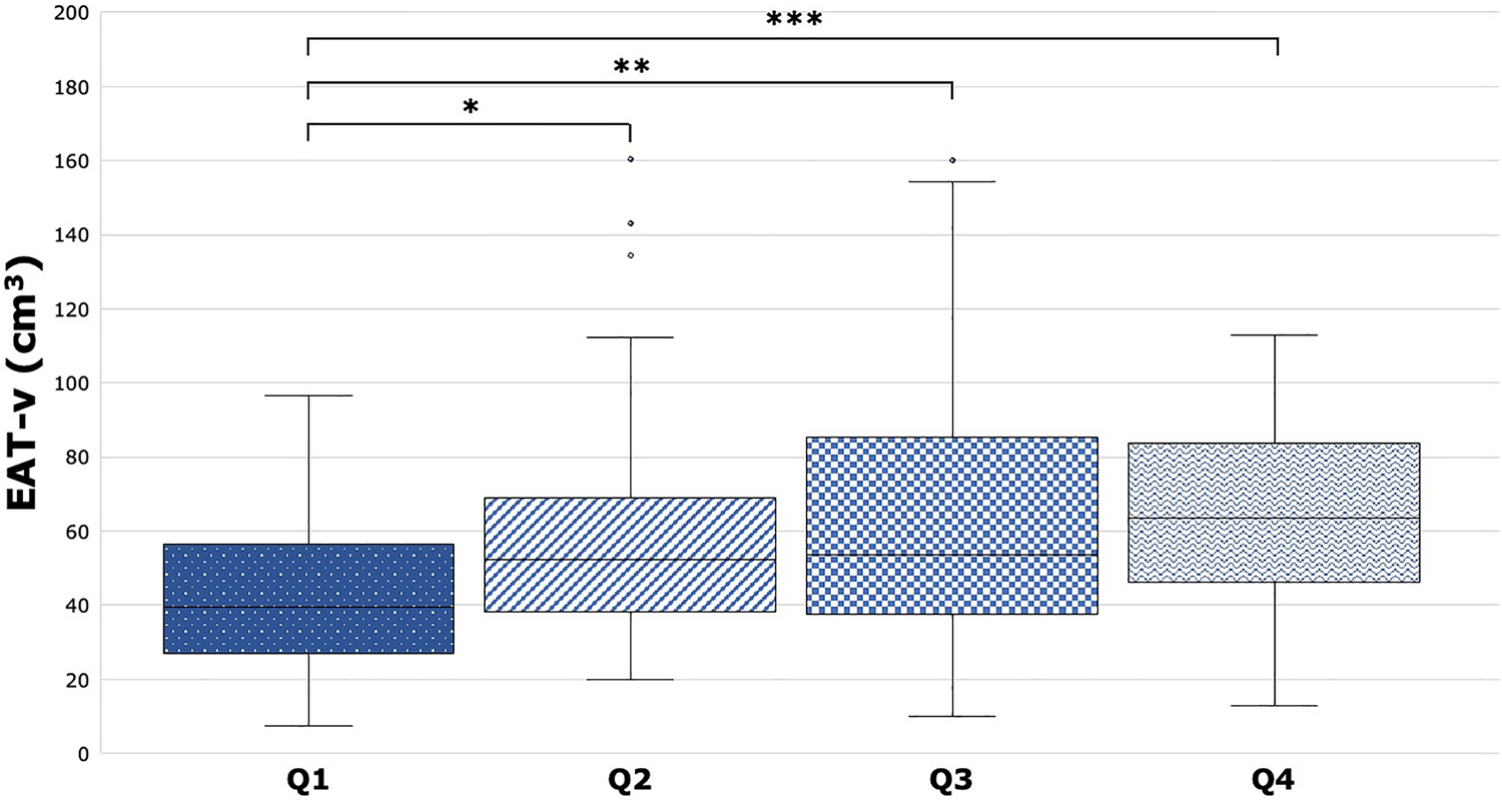
EAT-v across AIP quartiles. EAT-v progressively increased from AIP Quartile 1 to Quartile 4 (Q1: 39.4 [IQR 27.1–56.4] cm^3^; Q2: 52.3 [IQR 38.3–69.0] cm^3^; Q3: 53.7 [37.7–85.4] cm^3^; Q4: 63.5 [IQR 46.2–83.9] cm^3^). *p = 0.027; **p = 0.006; ***p = 0.011. *EAT-v* epicardial adipose tissue volume, *AIP* atherogenic index of plasma.

AIP was weakly correlated with EAT-v (r = 0.251, p = 0.001), and was associated with increased EAT-v on univariate linear regression analysis (OR 3.377 [95% CI 1.216–9.382], p = 0.020). This relationship continued to show significance when AIP was divided into quartiles; EAT-v progressively increased per quartile of AIP (Quartile 1: 39.4 [IQR 27.1–56.4] cm^3^; Quartile 2: 52.3 [IQR 38.3–69.0] cm^3^; Quartile 3: 53.7 [37.7–85.4] cm^3^; Quartile 4: 63.5 [IQR 46.2–83.9] cm^3^; p = 0.029; see Fig. 4), with significant pairwise differences between Quartile 1 and each subsequent quartile (all p < 0.05; see Fig. 4). AIP was not correlated with PCAT-a, PCAT-v or EAT-a, and there were no significant differences in these adipose tissue metrics across quartiles of AIP (all p > 0.05; see Supplementary Fig. S3–S5).

**Figure 5:**
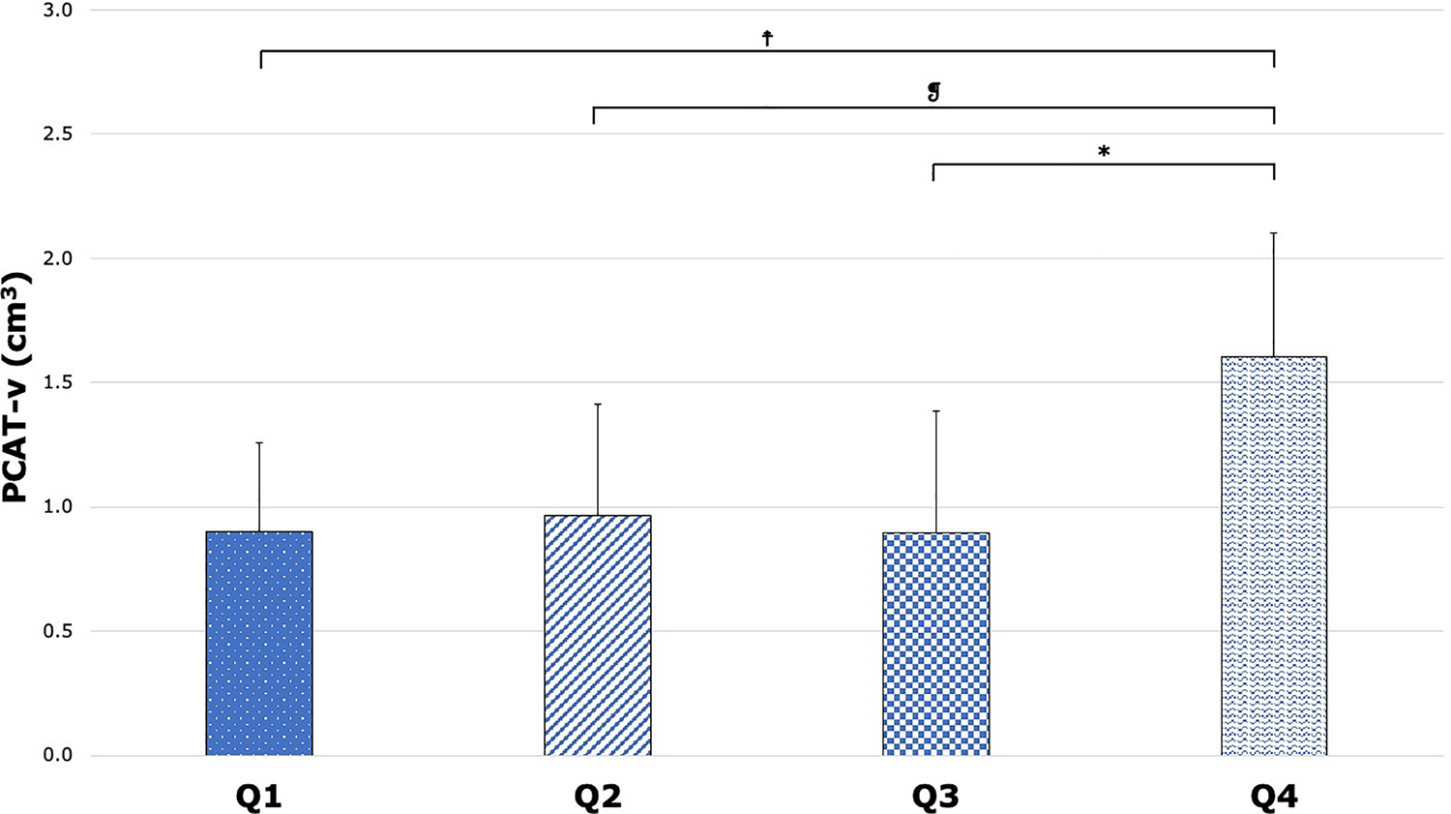
PCAT-v across AIP quartiles within a female-only subgroup. There was an overall significant difference across all quartiles in PCAT-v (Q1: 0.90 ± 0.36 cm^3^; Q2: 0.97 ± 0.45 cm^3^; Q3: 0.90 ± 0.49 cm^3^; Q4: 1.61 ± 0.50 cm^3^; p = 0.041). *p = 0.034; ^**☨**^p = 0.052; ^❡^p = 0.060. *PCAT-v* pericoronary adipose tissue volume, *AIP* atherogenic index of plasma.

To investigate a potential sex-specific effect in the relationship between AIP and adipose tissue markers, we evaluated changes to these metrics across AIP quartiles in male and female subgroups. There was no significant difference across AIP quartiles in EAT-v, EAT-a and PCAT-a within male and female subgroups (all p > 0.05; see Supplementary Figs. S5, S6, S8), and in PCAT-v within the male subgroup (p = 0.649; see Supplementary Fig. S7). A significant difference was found in PCAT-v was found within the female subgroup (p = 0.041), with a pairwise difference being found only between Quartile 3 and Quartile 4 (p = 0.034; see Fig. 5).

### Multivariable regression analyses

Multivariable analysis was performed to evaluate covariates associated with increased AIP. In the first model, EAT-v ≥ 52.3 cm^3^, age, traditional cardiovascular risk factors and plaque burden were included. In Model 1, increased AIP was associated with EAT-v ≥ 52.3 cm^3^, age, male sex and type II diabetes. In the second model, all covariates included in Model 1 were studied, but male sex was excluded from analysis. In Model 2, increased AIP was associated with EAT-v ≥ 52.3 cm^3^, age, type II diabetes and plaque burden (Table 2).

**Table 2:** Multivariable adjusted analyses of covariates associated with AIP. *AIP* atherogenic index of plasma, *EAT-v* epicardial adipose tissue volume, *IHD* ischaemic heart disease, *CT-LeSc* CT-Leaman score.

	Model 1			Model 2		
	Beta	95% CI	p value	Beta	95% CI	p value
EAT-v ≥ 52.3 cm^3^	**0.100**	**0.001, 0.198**	**0.047**	**0.117**	**0.019, 0.216**	**0.020**
Age	**− 0.005**	**− 0.009, − 0.001**	**0.018**	**− 0.005**	**− 0.009, − 0.001**	**0.007**
Male sex	**0.117**	**0.013, 0.221**	**0.027**	–	–	–
Obesity	0.039	**− **0.077, 0.155	0.509	0.021	**− **0.096, 0.138	0.722
Hypertension	0.008	**− **0.093, 0.109	0.880	0.000	**− **0.102, 0.102	0.997
Smoking	0.009	**− **0.093, 0.111	0.865	0.037	**− **0.063, 0.137	0.468
Family history IHD	**− **0.003	**− **0.101, 0.094	0.947	**− **0.010	**− **0.109, 0.089	0.839
Type II Diabetes	**0.165**	**0.031, 0.298**	**0.016**	**0.168**	**0.033, 0.304**	**0.015**
Plaque Burden	0.065	**− **0.012, 0.142	0.097	**0.088**	**0.014, 0.163**	**0.021**

Significant values are in bold.

Model 1 includes all traditional risk factors and Plaque Burden (No, Low and High plaque burden). Model 2 includes all traditional risk factors and plaque burden but excluding male sex.

Multivariable analysis was also performed to evaluate EAT-v ≥ 52.3 cm^3^ as a binary outcome variable. The first model included AIP, all traditional cardiovascular risk factors, and plaque burden. In Model 1, EAT-v ≥ 52.3 cm^3^ was associated with AIP, age and obesity. In the second model, all covariates included in Model 1 were studied, but male sex was excluded from analysis. In Model 2, AIP independently associated with EAT-v ≥ 52.3 cm^3^ (Table 3).

**Table 3:** Multivariable adjusted analyses of covariates associated with high EAT-v (≥ 52.3 cm^3^). *AIP* atherogenic index of plasma, *EAT-v* epicardial adipose tissue volume, *IHD* ischaemic heart disease, *CT-LeSc* CT-Leaman score.

	Model 1			Model 2		
	Exp(B)	95% CI	p value	Exp(B)	95% CI	p value
AIP	**3.581**	**1.084, 11.827**	**0.036**	**4.167**	**1.293, 13.435**	**0.017**
Age	**1.037**	**1.003, 1.072**	**0.033**	1.032	1.000, 1.065	0.050
Male sex	1.902	0.888, 4.075	0.098	–	–	–
Obesity	**2.413**	**1.042, 5.586**	**0.040**	2.184	0.958, 4.977	0.063
Hypertension	1.448	0.706, 2.970	0.312	1.369	0.674, 2.783	0.385
Smoking	1.108	0.532, 2.309	0.784	1.278	0.630, 2.591	0.497
Family history IHD	0.896	0.439, 1.827	0.762	0.849	0.421, 1.712	0.647
Type II Diabetes	0.755	0.289, 1.971	0.566	0.756	0.291, 1.963	0.565
Plaque Burden	0.988	0.561, 1.742	0.967	1.126	0.658, 1.928	0.664

Significant values are in bold.

Model 1 includes all traditional risk factors and Plaque Burden (No, Low and High plaque burden). Model 2 includes all traditional risk factors and plaque burden but excluding male sex.

We performed further adjusted analyses with an outcome of EAT-v ≥ 52.3 cm^3^ to evaluate isolated TG and HDL separately as potential predictors of increased EAT-v. The first model included either TG or HDL, all traditional risk factors, and plaque burden. In the second model, all covariates included in Model 1 were studied with the exception of male sex. TG was not associated with EAT-v ≥ 52.3 cm^3^ in either model. HDL was inversely associated with EAT-v ≥ 52.3 cm^3^ in conjunction with age and obesity in Model 1, and in Model 2 EAT-v ≥ 52.3 cm^3^ remained inversely associated with HDL and positively associated with age (Table 4).

**Table 4:** Multivariable adjusted analyses of covariates associated with high EAT-v (≥ 52.3 cm^3^). *AIP* atherogenic index of plasma, *EAT-v* epicardial adipose tissue volume, *IHD* ischaemic heart disease, *CT-LeSc* CT-Leaman score.

	Model 1			Model 2		
	Exp(B)	95% CI	p value	Exp(B)	95% CI	p value
HDL	**0.252**	**0.076, 0.836**	**0.024**	**0.203**	**0.065, 0.634**	**0.006**
Age	**1.034**	**1.001, 1.068**	**0.042**	**1.032**	**1.000, 1.065**	**0.049**
Male sex	1.555	0.709, 3.411	0.271	–	–	–
Obesity	**2.329**	**1.004, 5.402**	**0.049**	2.173	0.949, 4.974	0.066
Hypertension	1.218	0.585, 2.538	0.598	1.149	0.556, 2.371	0.708
Smoking	1.213	0.578, 2.548	0.610	1.341	0.655, 2.745	0.422
Family history IHD	0.823	0.403, 1.682	0.593	0.790	0.389, 1.601	0.513
Type II Diabetes	0.729	0.279, 1.906	0.519	0.713	0.274, 1.854	0.487
Plaque Burden	0.984	0.559, 1.734	0.957	1.053	0.609, 1.824	0.853

Significant values are in bold.

Model 1 includes all traditional risk factors and Plaque Burden (No, Low and High plaque burden). Model 2 includes all traditional risk factors and plaque burden but excluding male sex.

Finally, multivariable analysis was performed using AD as a binary outcome variable. The first model included EAT-v ≥ 52.3 cm^3^, all traditional cardiovascular risk factors, and plaque burden. In Model 1, AD was associated with age and male sex. In the second model, all covariates included in Model 1 were studied, but male sex was excluded from analysis. In Model 2, AD was associated with EAT-v ≥ 52.3 cm^3^ and age (Table 5).

**Table 5:** Multivariable adjusted analyses of covariates associated with AD. *AD* atherogenic dyslipidaemia, *EAT-v* epicardial adipose tissue volume, *IHD* ischaemic heart disease, *CT-LeSc* CT-Leaman score.

	Model 1			Model 2		
	Exp(B)	95% CI	p value	Exp(B)	95% CI	p value
EAT-v ≥ 52.3 cm^3^	2.292	0.951, 5.525	0.065	**2.560**	**1.085, 6.040**	**0.032**
Age	**0.964**	**0.930, 0.999**	**0.045**	**0.962**	**0.930, 0.994**	**0.021**
Male sex	**0.290**	**0.109, 0.772**	**0.013**	–	–	–
Obesity	1.188	0.446, 3.161	0.731	1.003	0.391, 2.571	0.996
Hypertension	1.404	0.558, 3.535	0.471	1.226	0.505, 2.977	0.653
Smoking	0.552	0.224, 1.363	0.198	0.756	0.325, 1.760	0.517
Family history IHD	1.535	0.656, 3.592	0.324	1.427	0.631, 3.226	0.394
Type II Diabetes	2.183	0.755, 6.313	0.150	2.105	0.745, 5.945	0.160
Plaque Burden	1.225	0.626, 2.398	0.554	1.528	0.804, 2.903	0.196

Significant values are in bold.

Model 1 includes all traditional risk factors and Plaque Burden (No, Low and High plaque burden). Model 2 includes all traditional risk factors and plaque burden but excluding male sex.

## Discussion

In our study, we report that AIP as a logarithmic and continuous representation of atherogenic dyslipidaemia (AD) is associated with increased EAT-v, as well as with age, male sex and type II diabetes after correction for traditional risk factors and coronary plaque burden. Furthermore, EAT-v increases incrementally across quartiles of AIP. Conversely, increased EAT-v is independently predicted by AIP. Male sex was a powerful predictor of increased AIP and EAT-v, but the relationship between AIP and EAT-v was present even after correction for this covariate. Finally, PCAT-a, a marker of coronary inflammation, was not significantly associated with AIP.

The combination of high TG with low HDL-c is associated with an increased risk of severe CAD and coronary events^7,16,17^, as well as with an adverse cardiometabolic profile including comorbidities such as obesity and type II diabetes^7,18–20^. In this regard, the logarithmic ratio of TG to HDL-c provides a holistic representation of the lipid interactions that characterise AD. In contrast, isolated lipid imbalances (i.e. only one of high TG or low HDL-c) may bear comparably less robust associations with cardiovascular risk^1,17^. Hypertriglyceridemia is often accompanied by decreased serum HDL, as HDL is more readily catabolised in its TG-rich form as it attempts to clear TG via reverse cholesterol transport^21^. A recent study^22^ found hypertriglyceridemia associated with both subclinical atherosclerosis and inflammation in non-coronary vessels, as shown through 18F-fluorodeoxyglucose (FDG) uptake on PET-CT. 18F-FDG uptake is reportedly higher within pericoronary fat^23^, however we found no difference in PCAT attenuation among subjects with elevated TG. It is well documented that high TG associates with coronary events after adjustment for traditional risk factors, but this becomes attenuated when adjusting for other parameters, such as HDL^24,25^. Decreased HDL, however, itself remained a significant predictor of CAD after adjustment for both traditional risk factors and lipid levels^25^. In our study, we found only EAT volume correlated with both increased TG and decreased HDL independently, and that HDL, but not TG, inversely predicted increased EAT-v on multivariable analysis. It is unclear if increased TG and decreased HDL precedes or is causal to the accumulation of EAT, though numerous observational studies have nevertheless highlighted a relationship between the two variables. One study found that EAT volume indexed to total body volume on CCTA was positively associated with TG and with markers of proatherogenic HDL characteristics, and after adjustment for traditional risk factors apoA-II content in HDL (characteristically upregulated in proatherogenic HDL) was among age and waist circumference in predicting increased EAT^26^. Goeller et al.^11^ reported that EAT-v was associated with increased TG and decreased HDL, as well as inflammatory biomarkers in plasminogen-activator inhibitor-1 (PAI-1) and monocyte chemoattractant protein-1 (MCP-1), highlighting a complex and multifaceted relationship between EAT, vascular inflammation, and dyslipidaemia.

In describing the log-transformed ratio of TG to HDL, AIP provides an alternative representation of dyslipidaemia as a normally distributed continuous covariate. AIP is a quantitative metric, describing the numerical ratio in TG and HDL concentrations rather than phenotypic characteristics of these biomolecules. In comparison, the “quality” of lipid particles such as HDL may provide further atherogenic contributions, and while this remains beyond the scope of AIP, this index continues to demonstrate utility in providing additive value to LDL in cardiovascular risk prediction, as shown by increased prevalence of ischaemic heart disease in patients with low LDL but an adverse TG to HDL balance^7^. Furthermore, AIP predicts mortality endpoints after adjustment for traditional risk factors and other lipid parameters, such as TC and LDL^27,28^. Moreover, increases in the TG/HDL ratio may accompany an increase in small dense LDL molecules that is associated with enhanced atherogenicity^29,30^. We found AIP did not correlate with LDL (p = 0.623), but was positively correlated with coronary plaque burden as shown through CT-LeSc, in agreement with previous studies that have used alternative methods of assessing CAD severity^6,31^. Further, we report that AIP was associated with increased EAT-v, and that EAT-v increases incrementally across quartiles of AIP. As discussed, increased EAT-v is linked with adverse lipid and inflammatory changes^11,26^, and has also been strongly associated with MACE^32^. Similarly, increased AIP accompanies vascular inflammatory markers such as neutrophil–lymphocyte ratio (NLR in patients with acute coronary syndrome (ACS)^27,28,33–35^. The relationship between AIP and EAT in our study highlights that these variables are not simply disparate markers of coronary risk but are likely interrelated in contributing to adverse coronary outcomes. Previously, Erdur et al.^36^ found a correlation between EAT and AIP among patients with end-stage renal disease, but this association was diminished after adjustment for clinical covariates. Similarly, Akbas et al.^37^ found increased two-dimensional EAT-thickness, quantified on echocardiography, was associated with NLR, BMI and AIP, but multivariable analysis rendered the relationship between EAT and AIP statistically insignificant. Notably, these studies did not adjust for male sex or coronary plaque burden, which may be powerful covariates associated with both EAT and AIP. In contrast, our adjusted analyses highlighted that AIP does remain significantly associated with EAT volume even after correction for plaque burden, male sex and traditional risk factors, but interestingly, this relationship is strengthened significantly in models that excluded male sex as a covariate.

Growing evidence highlights a potentially significant impact of sex on AIP. Cai et al.^38^ found that among a Chinese study population of subjects aged ≤ 35 years, the relationship between ACS and increased AIP was present only in men. Likewise, Ni et al.^39^ reported an association of AIP in men, but not in women, with the presence of CAD. Interestingly, a propensity-matched study in a postmenopausal female cohort found AIP did indeed predict CAD presence after adjustment for traditional risk factors^40^. This observation echoes the robust association of EAT-v with CAD in male subjects^41,42^, and the increased EAT mass observed in postmenopausal compared to premenopausal women^42,43^. While we did not control for factors pertaining to menopause, our adjusted analysis revealed that among all traditional risk factors, both AIP and EAT-v remained associated with each other, but the effect of male sex attenuated this association. Indeed, AIP was an independent predictor of increased EAT volume, but only in a model in which male sex was excluded. Conversely, EAT volume associated with increased AIP in adjusted analysis including and excluding male sex, but in the latter model the relationship was strongest. Such is not only consistent with previous findings but suggests future studies into the relationship between dyslipidaemia and EAT would need to sufficiently account for the influence of gender.

While EAT is a well-established marker of adverse cardiac risk, PCAT attenuation (PCAT-a) presents an alternative imaging biomarker that more precisely reflects coronary inflammation. PCAT-a has been associated with inflammatory biomarkers in vivo^10^ and is also associated with characteristics of vulnerable atherosclerotic plaque^44–47^. There was no significant difference in PCAT-a between AD and non-AD patients, nor did we find any relationship between PCAT-a and AIP. In contrast, Patil et al.^48^ recently reported that the ratio of TG to HDL was a predictor of increased cardiac uptake of 18F-NaF on PET-CT upon adjustment for numerous covariates including age, gender, fasting glucose and lipids, providing strong evidence from an imaging standpoint of the link between dyslipidaemia and coronary inflammation. A potential consideration for why we found no relationship between PCAT-a and dyslipidaemia in our study may be that a significant proportion of our patients were on statin therapy. At least a third of patients in both AD and non-AD groups were on statins at the time of CCTA (42.9% vs. 33.8%; Table 1), and while there was no difference in the prevalence of statin use between these groups (p = 0.447), this may still have diminished a relationship, if any, between dyslipidaemia and PCAT-a. Ultimately, our study was not designed with the primary aim of evaluating the impact of statin use and thus these findings must be interpreted cautiously.

EAT volume, therefore, is associated with AIP after adjustment for traditional risk factors, further confirming the profound effects of AD and metabolic syndrome on cardiovascular disease. The accumulation of epicardial adiposity and AIP have independently been established as markers of coronary risk, including increased plaque burden, vulnerable plaque features and MACE^11,12,27,28,32^. While previous studies espouse a limited relationship between EAT and AIP after adjustment for other clinical variables, we found that EAT-v and AIP do indeed associate with one another in an adjusted model including coronary plaque burden, male sex and traditional risk factors. These metrics may therefore be additive and superior to individual lipid parameters in their capacity to evaluate and prognosticate coronary risk, although future studies would need to perform adjusted analysis of both EAT and AIP concurrently within the same cohort to further investigate this relationship.

Our study has several limitations. As the study cohort was small and derived retrospectively, the impact of selection bias and a limited generalisability to the wider population cannot be excluded. Moreover, the application of our inclusion and exclusion criteria in addition to retrospective sampling left us with a comparatively smaller group of patients with AD. The absence of outcome data prevented us from assessing time-adjusted effect of AD markers on coronary events. Moreover, a proportion of both AD and non-AD subgroups within our study were undergoing statin therapy at the time of CCTA, and we did not control for its lipid-lowering and anti-inflammatory effects in our analysis. Finally, the duration of time between CCTA and date of lipid collection was varied, which may have impacted our analysis on isolated lipid parameters. Nevertheless, we were still able to report AIP was associated with EAT-v even after multivariable adjustment, although future studies might further validate our findings by using lipid values that were obtained in a timelier manner.

AIP as a marker of dyslipidaemia is associated with increased EAT volume when adjusted for coronary plaque burden and traditional cardiovascular risk factors. However, PCAT-a, a marker of coronary inflammation, is not significantly associated with AD. These findings suggest that atherogenic dyslipidaemia is associated with an adverse adipose tissue phenotype indicative of increased metabolic and coronary risk, but larger studies are required to validate these findings.

## Methods

### Study population

We retrospectively obtained a cohort of 211 patients underwent clinically indicated coronary CTA at MonashHeart and had available lipid data between 2008 and 2011. Fifty patients were excluded on the basis of incomplete lipid data, a duration of ≥ 12 months between CCTA and lipid collection, and poor image quality as adjudicated on a five-point Likert scale^49^. Cardiovascular risk factors, BMI, and statin history were all obtained at the time of CCTA via patient questionnaires. Hypertension was defined as systolic blood pressure of > 140 mmHg or diastolic blood pressure of > 90 mmHg at the time of CCTA or the diagnosis/treatment of hypertension. A positive smoking history was defined by self-reporting and included both current and ex-smokers. A positive family history of ischaemic heart disease (IHD) was defined as presentation of any immediate family member with IHD before the age of 60. Type II diabetes mellitus was defined as HbA1c ≥ 6.5% or treatment with glucose-lowering medications. Height and weight were also obtained at the time of CCTA, and body mass index (BMI) was calculated from these parameters (kg/m^2^). Obesity was defined as BMI ≥ 30 kg/m^2^. Statin-positive patients were classified as those already on any statin therapy (atorvastatin, pravastatin, rosuvastatin, simvastatin) at the time of CCTA.

The study was approved by the Monash Health Human Research Ethics Committee (No. EC00382) and the study in its entirety was performed in accordance with all relevant guidelines and regulations. Consent was waived for our study cohort by the Monash Health Human Research Ethics Committee due to the retrospective nature of our analysis.

### Coronary CTA protocol

CCTA acquisitions were obtained using a 320-detector row scanner (Aquilion Vision; Canon Medical Systems Corporation, Otawara, Japan) at MonashHeart, or a 128-detector-row scanner (Somatom Definition, Siemens Medical, Erlangen, Germany) at Mildura Base Hospital. All scans were performed using retrospective ECG-gating during a single-breath hold. Beta-blockers were administered to maintain a heart rate of 60 bpm. All scans were contrast-enhanced through use of Omnipaque 350 (60–90 mL) administered intravenously at a rate of 5 mL/s. Acquisition parameters adhered to a limited range for all patients in our cohort: 300–500 mA tube current, 100–120 kV tube voltage, collimation 320 × 0.5 mm, gantry rotation time 275 ms and temporal resolution 175 ms. Images were collated and reconstructed using a reconstruction kernel (FC03). All scans were performed in conjunction with department-specific protocol and other relevant guidelines^49,50^.

### Coronary artery disease classification and quantification

All CCTA scans were evaluated by two cardiologists concomitantly according to a 16-segment model as per clinical guidelines^51^. Coronary segments that were > 2 mm in diameter were assessed for plaque presence and composition, and CAD was classified as the presence of at least one plaque in the coronary vessels irrespective of stenosis. The CT-Leaman Score (CT-LeSc) was used to numerically quantify an adjusted value for plaque burden, accounting for stenosis (obstructive versus non-obstructive), plaque morphology (calcified versus non-calcified or mixed), vessel localisation, and vessel dominance^52^. Three categories of plaque burden were studied based on previously defined thresholds: no coronary plaque was defined as CT-LeSc = 0, low plaque burden as CT-LeSc = 0.01–8.29, and high plaque burden as CT-LeSc ≥ 8.3^52^.

### Lipid analysis

All lipid profiles were obtained at one of two laboratories (Monash Pathology, Victoria; Barratt & Smith Pathology, Victoria) after an 8–12 h fast. Lipids obtained were total cholesterol (TC), triglyceride (TG), high-density lipoprotein cholesterol (HDL-c) and low-density lipoprotein (LDL-c) cholesterol. TC, TG and HDL-c levels were assessed via standard enzymatic assays (Beckman Coulter, California, USA). LDL-c were measured either directly in a separate laboratory (Dorevitch Pathology, Victoria) via assay (Roche Diagnostics, Indianapolis, USA), or calculated via the Friedewald equation when TG ≤ 4.5 mmol/L. Lipid thresholds were defined according to the Third Report of the National Cholesterol Education (NCEP), Adult Treatment Panel III: high TC was defined as serum ≥ 5.2 mmol/L, high TG as ≥ 1.7 mmol/L, low HDL as < 1.0 mmol/L (men) or < 1.3 mmol/L (women), and high LDL ≥ 3.4 mmol/L^53^. AD was defined as the presence of both high TG and low HDL-c according to these criteria^53^. AIP was defined as the base 10 logarithmic ratio of TG to HDL: log_10_ (TG/HDL)^3^.

### Adipose tissue segmentation

Adipose tissue was quantified on routine coronary CTA images, and was defined as all three-dimensional voxels ranging from − 190 to − 30 Hounsfield Units (HU) within a volume of interest appropriate to the depot studied. Only adipose tissue within the pericardial sac was studied and consisted of epicardial adipose tissue (EAT) and per coronary adipose tissue (PCAT). Adipose tissue volume was measured in cm^3^ and attenuation was measured in Hounsfield Units (HU) and all analyses were performed by trained operators with at least one-year experience with the software (JY, MI, ZC, EL).

EAT was quantified on a semi-automated basis using QFAT (version 2.5, Cedars-Sinai Medical Center, Los Angeles, CA, USA). The operator first delineated the visceral pericardium in 5–7 cardiac slices ranging from the pulmonary bifurcation to the posterior descending artery^54^. The software then automatically computes adipose tissue within this operator-defined volume, determining total EAT-volume (EAT-v) and mean EAT attenuation (EAT-a) per patient. PCAT was segmented using AutoPlaque (version 2.5, Cedars-Sinai Medical Center, Los Angeles, CA, USA). The operator identified the right coronary artery using a manually-traced centreline within the vessel lumen, then delineated the adventitia of a 40 mm longitudinal segment, commencing 10 mm from the ostium to exclude potential image artefacts arising from the aortic wall^10^. After this segment was defined, the software then computes total PCAT-volume (PCAT-v) and mean PCAT attenuation (PCAT-a) for this segment within a radial distance from the vessel wall of 3 mm, corresponding to the typical diameter of the RCA^10^.

### Statistical analysis

Data was described by mean and standard deviation for parametric, normally distributed data, and by median and interquartile range (IQR) for non-parametric data. EAT-v and EAT-a were compared between groups using the Mann–Whitney U test, while PCAT-v and PCAT-a were compared between groups using the independent samples *t*-test. The relationship between adipose tissue metrics (EAT-v, EAT-a, PCAT-v, and PCAT-a) and AIP, as well as individual lipid parameters (TC, TG, HDL, LDL) were studied using either Pearson or Spearman correlation analysis. Significant correlations were further explored in univariable linear regression. Regarding cardiovascular risk factors, age and BMI were compared between groups using independent samples *t*-test and Mann–Whitney U test, respectively; categorical risk factors (male sex, obesity, hypertension, smoking, family history IHD, statin use) were studied using chi-square analysis. The relationship between AIP and risk factors was likewise studied using correlation and univariable linear regression analysis and independent samples *t*-test. The one-way ANOVA test or Kruskal–Wallis test was used to study differences in adipose tissue metrics across AIP quartiles and categories of plaque burden. All covariates achieving p < 0.2 were included in further multivariable analysis. Two multivariable models were used: Model 1 included age, male sex, obesity, hypertension, smoking, family history of IHD, type II diabetes, plaque burden as a categorical covariate, and any other covariates that achieved significance on univariable analysis. Model 2 included all covariates included in Model 1, except for male sex. For all analysis, a two-sided p-value of p < 0.05 was considered significant. All statistical analysis was performed using SPSS (version 27).

## Supplementary Information

Supplementary Information.

## Data Availability

The datasets analysed during the current study are not publicly available due to patient confidentiality, as said data was obtained for clinical purposes. All inquiries regarding access to study data may be directed to DW.
