# Supplementary material for: Atherogenic index of plasma is associated with epicardial adipose tissue volume assessed on coronary computed tomography angiography
Source: Sci Rep. 2022 Jun 10;12:9626. doi: 10.1038/s41598-022-13479-5 (PMC9187675; doi:10.1038/s41598-022-13479-5)
Supplement: Supplementary file 1 — Supplementary Information. [file 41598_2022_13479_MOESM1_ESM.docx]

***SUPPLEMENTARY FIGURES***


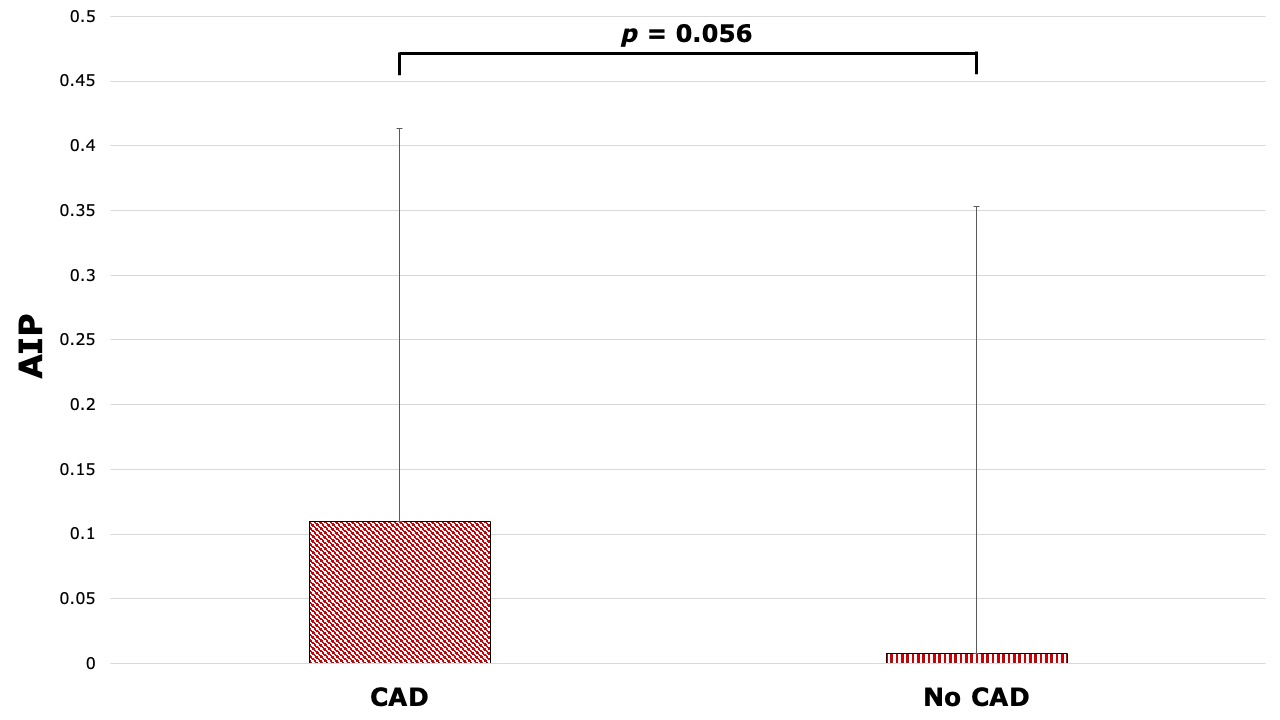


**Figure S1**. AIP in patients with CAD vs. patients with no CAD (0.113±0.304 vs. 0.008±0.345, p=0.056). *AIP: atherogenic index of plasma; CAD: coronary artery disease*.


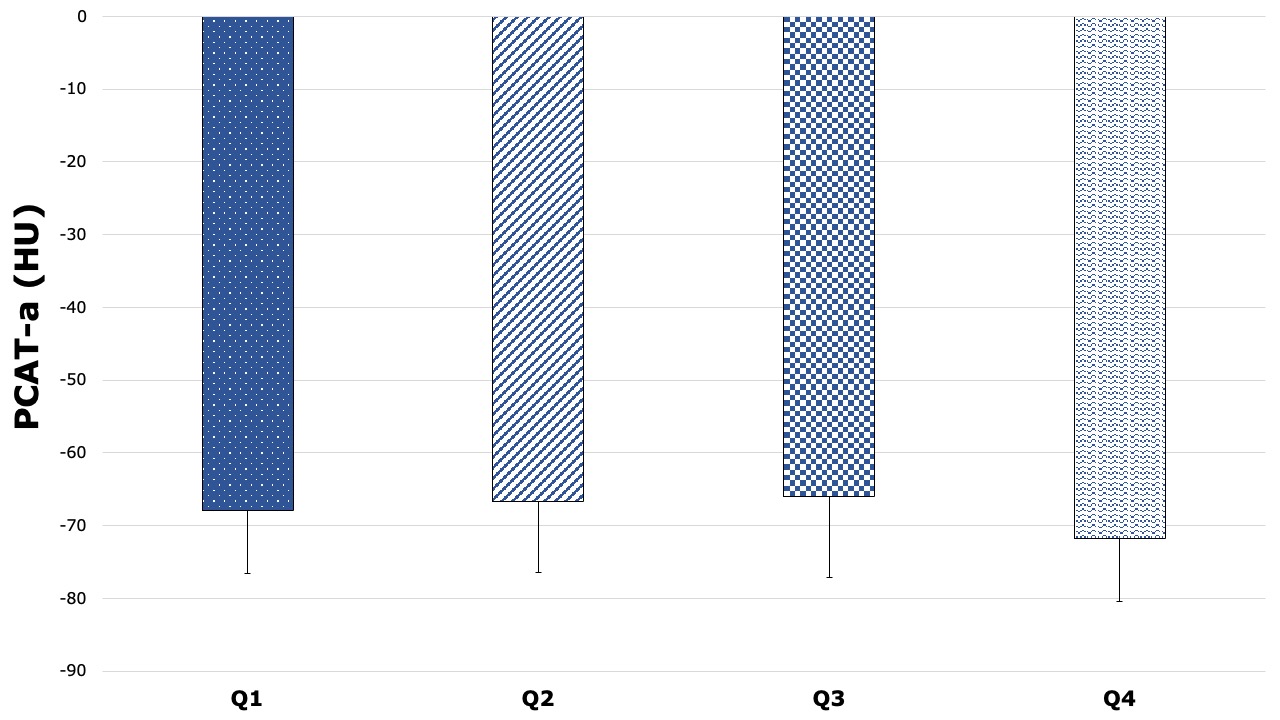


**Figure S2.** PCAT-a across quartiles of AIP (Q1:-68.0±8.6 HU; Q2: -66.7±9.8 HU; Q3: -66.1±11.1 HU; Q4: -71.8±8.6 HU; p=0.400). *PCAT-a: pericoronary adipose tissue attenuation; AIP: atherogenic index of plasma; HU: Hounsfield units.*


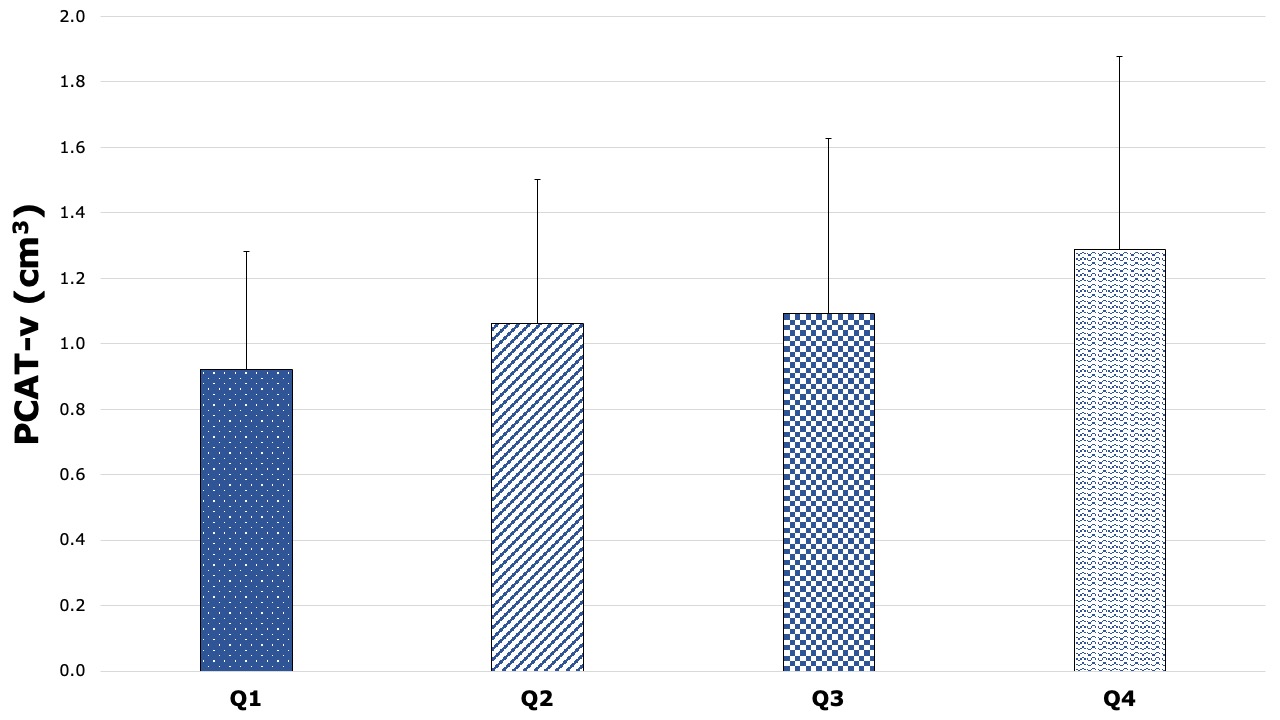


**Figure S3.** PCAT-v across quartiles of AIP (Q1: 0.92±0.36cm^3^; Q2: 1.06±0.44cm^3^; Q3: 1.09±0.53cm^3^; Q4: 1.29±0.59cm^3^; p=0.270). *PCAT-v: pericoronary adipose tissue volume; AIP: atherogenic index of plasma.*


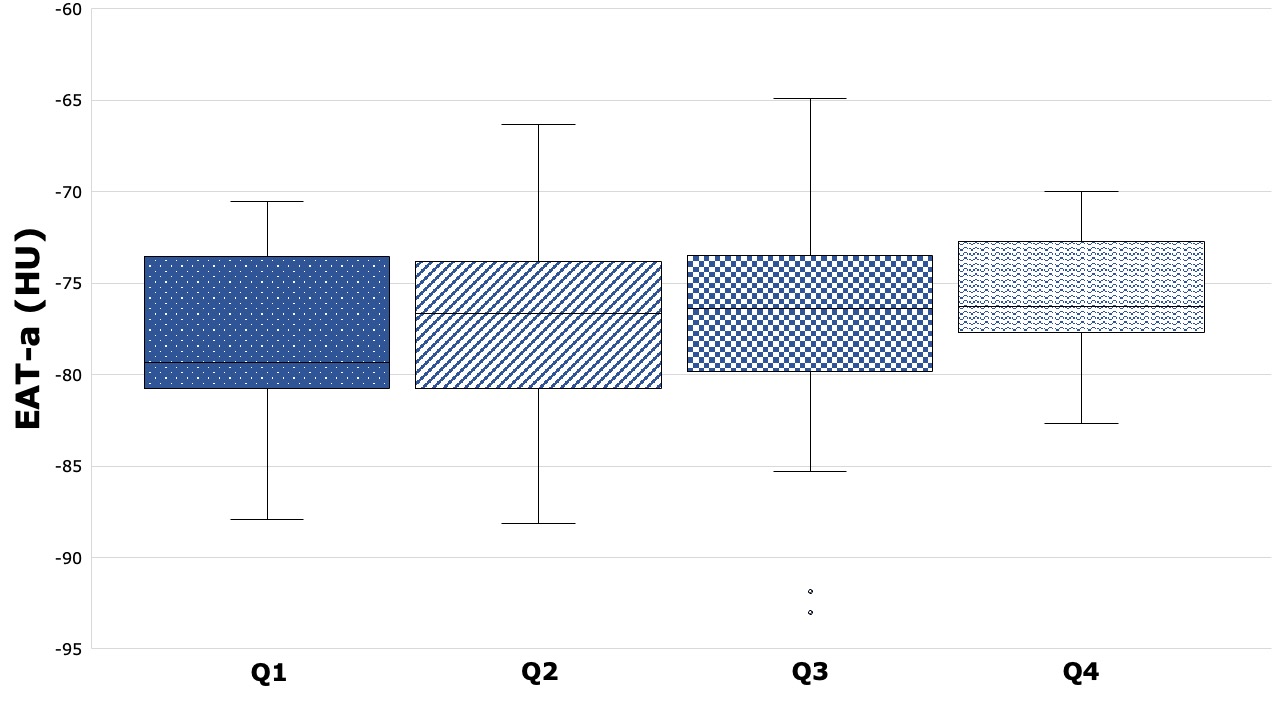


**Figure S4**. EAT-a across AIP quartiles (Q1: -79.3 [IQR -80.8 to -73.5] HU; Q2: -76.7 [IQR -80.8 to -73.8] HU; Q3: -76.4 [IQR -79.8 to -73.5] HU; Q4 -76.3 [IQR -77.7 to -72.7] HU; p=0.402). *EAT-a: epicardial adipose tissue attenuation; AIP: atherogenic index of plasma; IQR: interquartile range; HU: Hounsfield units.*


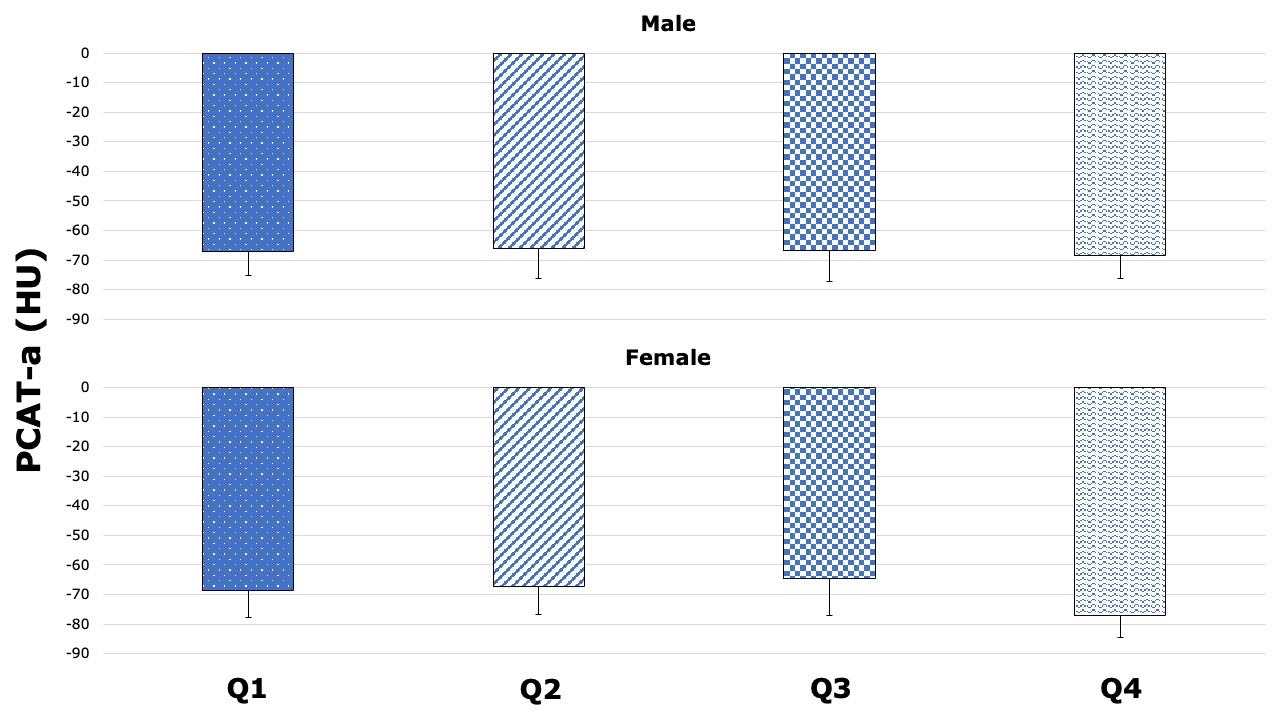


**Figure S5**. PCAT-a across AIP quartiles in male (Q1: -67.1±8.1 HU; Q2: -66.2±10.1 HU; Q3: -66.9±10.4 HU; Q4: -68.3±7.9 HU; p=0.968) and female subgroups (Q1: -68.5±9.3 HU; Q2: -67.2±9.6 HU; Q3: -64.7±12.4 HU; Q4: -77.1±7.4 HU; p=0.191). *PCAT-a: pericoronary adipose tissue attenuation; AIP: atherogenic index of plasma; HU: Hounsfield units.*


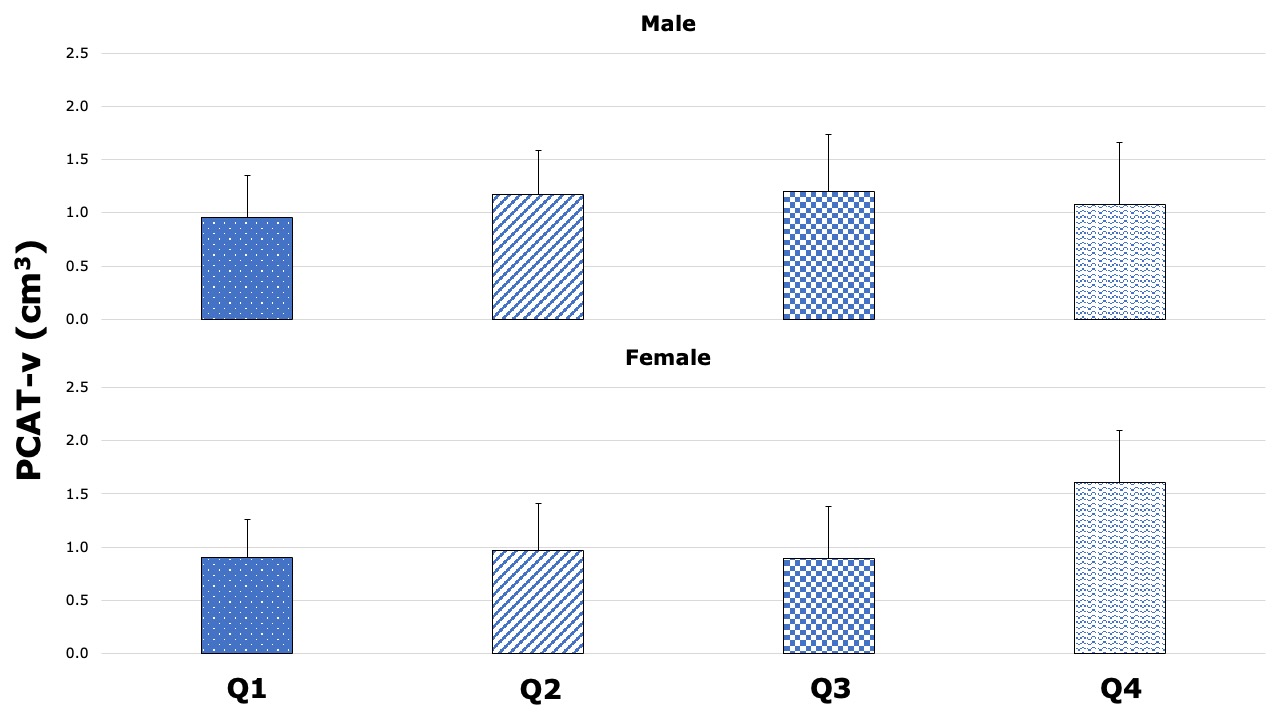


**Figure S6.** PCAT-v across AIP quartiles in male (Q1: 0.96±0.39 cm^3^; Q2: 1.17±0.41 cm^3^; Q3: 1.20±0.53 cm^3^; 1.08±0.59 cm^3^; p=0.649) and female subgroups (Q1: 0.90±0.36 cm^3^; Q2: 0.90±0.45 cm^3^; Q3: 0.90±0.49 cm^3^; Q4: 1.61±0.50 cm^3^; p=0.041). *PCAT-v: pericoronary adipose tissue volume; AIP: atherogenic index of plasma*.


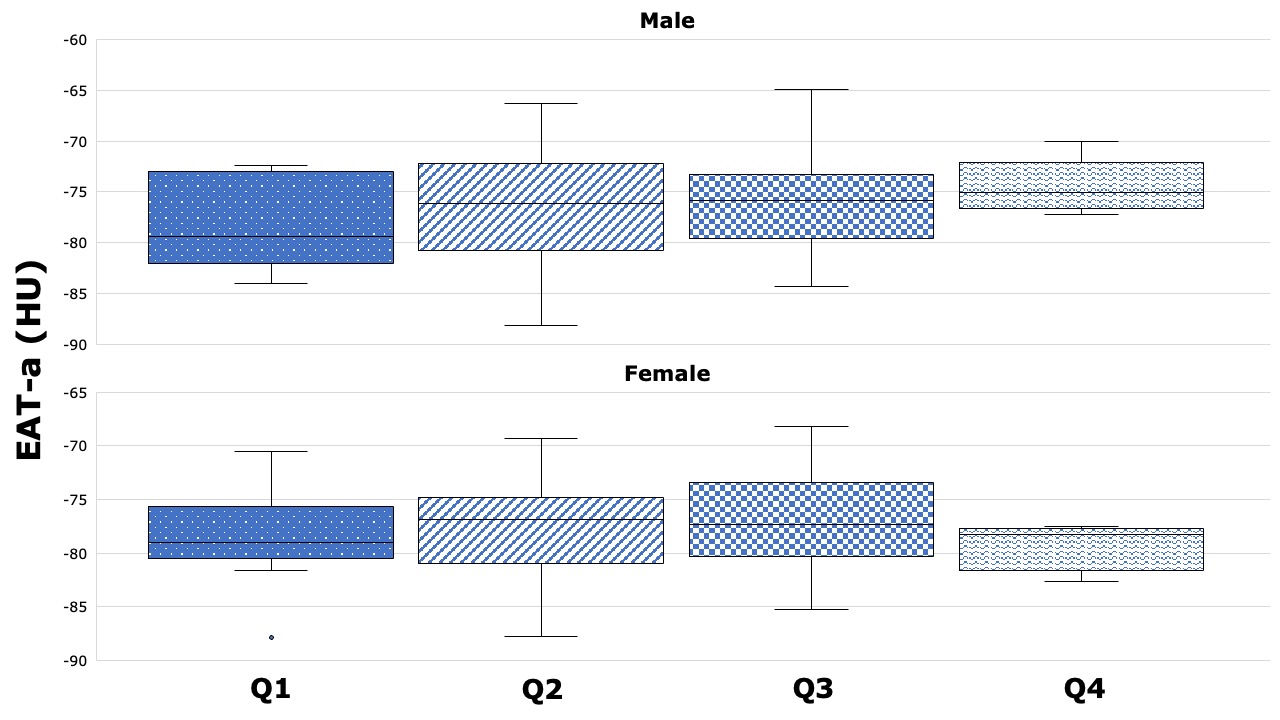


**Figure S7.** EAT-a across AIP quartiles in male (Q1: -79.3 [IQR -82.1 to -73.0] HU; Q2: -76.2 [IQR -80.8 to -72.3] HU; Q3: -75.9 [IQR -79.6 to -73.3] HU; Q4: -75.1 [IQR -76.6 to -72.1] HU; p=0.548) and female subgroups (Q1: -79.1 [IQR -80.5 to -75.7] HU; Q2: -76.9 [IQR -81.0 to -74.9] HU; Q3: 77.3 [IQR -80.3 to -73.5] HU; Q4: -78.3 [IQR -81.6 to -77.7] HU; p=0.705). *EAT-a: epicardial adipose tissue attenuation; AIP: atherogenic index of plasma; IQR: interquartile range; HU: Hounsfield units.*


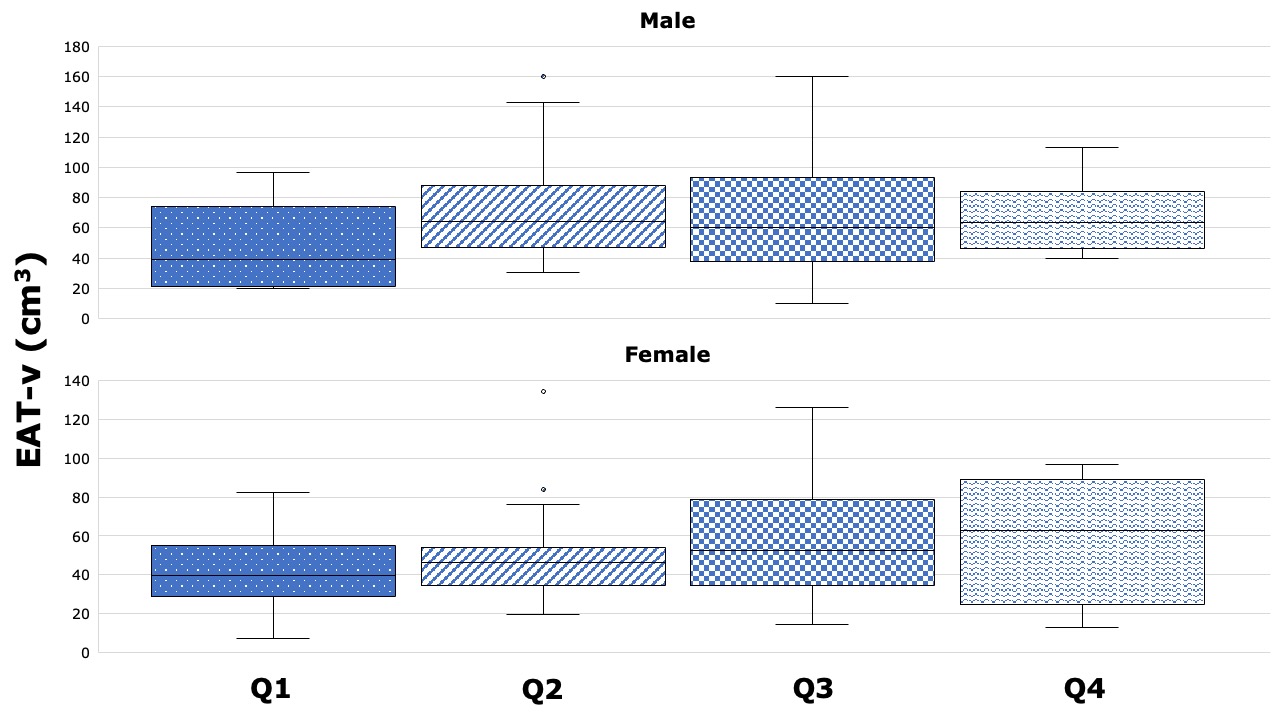


**Figure S8.** EAT-v across AIP quartiles in male (Q1: 39.4 [IQR 21.3 to 74.0] cm^3^; Q2: 64.3 [IQR 46.8 to 88.0] cm^3^; Q3: 60.2 [IQR 38.1 to 93.0] cm^3^; Q4: 63.5 [IQR 46.2 to 83.9] cm^3^; p=0.267) and female subgroups (Q1: 39.9 [IQR 28.7 to 55.3] cm^3^; Q2: 46.4 [IQR 34.8 to 54.1] cm^3^; Q3: 52.3 [IQR 34.8 to 78.9] cm^3^; Q4: 62.7 [IQR 24.7 to 88.9] cm^3^; p=0.315). *EAT-v: epicardial adipose tissue volume; AIP: atherogenic index of plasma*; *IQR: interquartile range.*
